# Validation of a Clinical-Radiographic Score to Assess the Probability of Pulmonary Tuberculosis in Suspect Patients with Negative Sputum Smears

**DOI:** 10.1371/journal.pone.0018486

**Published:** 2011-04-05

**Authors:** Alonso Soto, Lely Solari, Javier Díaz, Alberto Mantilla, Francine Matthys, Patrick van der Stuyft

**Affiliations:** 1 Department of Medicine, Hospital Nacional Hipólito Unanue, Lima, Peru; 2 Epidemiology and Disease Control Unit, Institute of Tropical Medicine, Antwerp, Belgium; 3 Department of Medicine, Hospital Nacional Cayetano Heredia, Lima, Peru; McGill University, Canada

## Abstract

**Background:**

Clinical suspects of pulmonary tuberculosis in which the sputum smears are negative for acid fast bacilli represent a diagnostic challenge in resource constrained settings. Our objective was to validate an existing clinical-radiographic score that assessed the probability of smear-negative pulmonary tuberculosis (SNPT) in high incidence settings in Peru.

**Methodology/Principal Findings:**

We included in two referral hospitals in Lima patients with clinical suspicion of pulmonary tuberculosis and two or more negative sputum smears. Using a published but not externally validated score, patients were classified as having low, intermediate or high probability of pulmonary tuberculosis. The reference standard for the diagnosis of tuberculosis was a positive sputum culture in at least one of 2 liquid (MGIT or Middlebrook 7H9) and 1 solid (Ogawa) media. Prevalence of tuberculosis was calculated in each of the three probability groups.

684 patients were included. 184 (27.8%) had a diagnosis of pulmonary tuberculosis. The score did not perform well in patients with a previous history of pulmonary tuberculosis. In patients without, the prevalence of tuberculosis was 5.1%, 31.7% and 72% in the low, intermediate and high probability group respectively. The area under de ROC curve was 0.76 (95% CI 0.72–0.80) and scores ≥6 had a positive LR of 10.9.

**Conclusions/Significance:**

In smear negative suspects without previous history of tuberculosis, the clinical-radiographic score can be used as a tool to assess the probability of pulmonary tuberculosis and to guide the decision to initiate or defer treatment or to requesting additional tests.

## Introduction

In most low income countries, the diagnosis of pulmonary tuberculosis continues to rely on the search for Acid-Fast Bacilli (AFB) in sputum smears, which has a sensitivity between 50 and 80% [1]–[3]. This sensitivity varies according to factors such as the number of smears examined, the compliance with laboratory procedure guidelines, the training of personnel and patients characteristics, including their HIV status [4]. Clinical suspects of pulmonary tuberculosis (PTB) with negative sputum smears constitute a diagnostic challenge, as many of them would result positive if assessed with culture. Furthermore, smear negative pulmonary tuberculosis (SNPT) represents an important public health problem in many countries, with an estimated proportion of between 30–60% of all tuberculosis cases, according to the setting [1], [3], [5]–[7], and a mortality up to 25% [8] in populations with high prevalence of HIV infection.

Access to culture is limited in many high tuberculosis incidence environments. Furthermore, classical culture in solid media has the important drawback of the time required before obtaining results, which takes up to 6 weeks. Liquid media can significantly shorten the diagnostic delay, but they are unaffordable for laboratories in resource constrained settings. New molecular techniques are also expensive, possibly only marginally helpful for smear negative cases and their utility at a programmatic level is still controversial [9].

The utility of exhaustive clinical evaluation of SNPT suspects has not often been investigated in depth and in day to day practice signs and symptoms are only being used according to the criteria of the treating physician. Moreover, the clinical definitions in existing guidelines are rather vague [10]–[12] and do not allow to classify patients according to their probability of having tuberculosis. Some diagnostic algorithms and clinical decision rules for assessing the probability of PTB among smear-negative suspects have been proposed, but they have generally not been validated in external populations [13]–[16]. We have previously developed in Lima, Peru, a Clinical prediction rule in the form of a “score” for assigning the probability of having SNPT in patients with clinical suspicion of PTB and negative sputum smears [17]. Based on our results, we recommended its use to classify patients as having low, intermediate or high probability of SNPT, in order to guide the decision for intensified diagnostic workup. The purpose of the present study was to prospectively assess the validity of this score in an independent patient sample.

## Methods

### Objective

To validate a clinical-radiographic score that assess the probability of pulmonary tuberculosis in suspect patients with negative sputum smears in high incidence settings with limited resources.

### Participants

This study was conducted in Cayetano Heredia and Hipólito Unanue Hospitals, two university-affiliated hospitals with a catchment population of about two million people in Lima, Peru. The area has a high incidence of tuberculosis (151/100000 population [18]) and a concentrated HIV/AIDS epidemic (prevalence of less than 1 percent in general population [19]).All consecutive adult patients, whether referred, self referred or directly consulting that presented between September 2005 and March 2008 in the departments of internal and pulmonary medicine of the 2 hospitals with suspicion of PTB were evaluated for eligibility.

Clinical suspicion of PTB was defined as the presence of cough for at least 2 weeks and any of the following: fever, weight loss or dyspnoea. PTB suspects were included after at least two negative Acid Fast Bacilli (AFB) smears in conventional sputum specimens either at a first line health facility or at the hospital level. Patients diagnosed at that point with obvious other respiratory conditions (e.g. asthmatic crises or COPD exacerbation) were excluded.

### Description of Clinical and Laboratory procedures

At intake we collected information on the sociodemographic and clinical characteristics of all participants in face to face interviews.

We performed a chest X ray on each participant that was read by the general practitioner (GP) in charge of the recruitment of patients in each of the participating hospitals. They assessed specifically the presence or absence of apical and/or miliary infiltrates. The readings were blindly revised by a TB specialist in the same hospital, and disagreement was resolved by an experienced radiologist.

An additional sputum sample was taken for a concentrated sputum smear (prepared after decontamination using 4% NaOH and subsequent centrifugation) and cultures in Ogawa, Middlebrook 7H9 and Mycobacterial Growth Indicator tube (MGIT). Laboratory personnel was blinded to the clinical history of the patient. Voluntary HIV testing was performed after counselling. Diagnostic work up for alternative diagnosis, if necessary, fell under the responsibility of the treating physician and documenting this process and its outcome was not a study objective.

### Clinical score

The previously developed score was derived using logistic regression, assigning points to each of the predictive findings included in the model according to the strength and direction of the association with smear negative TB [17], and optimal cut-off points were identified with ROC analysis. It includes, in brief, four clinical variables: age more than 45 years (-1 point), haemoptysis (2 points), weight loss (1 point) and expectoration (-1 point), and two radiographic variables: apical infiltrate (3 points) and miliary infiltrate (4 points). The total scores range from −2 to 10 points. A patient is classified as having low (negative scores), intermediate (0 to 4 points) or high probability of tuberculosis (more than 4 points). The personnel classifying the patients' probabilities were blinded to the laboratory results and vice versa.

### Ethics

The study was conducted inside an ongoing research project aiming at the development of a comprehensive clinical approach towards the diagnosis of smear negative tuberculosis in Peru. It was approved by the ethics committee of Universidad Peruana Cayetano Heredia. Written informed consent was obtained from all patients.

### Statistical methods

SNPT was defined as a positive result in the concentrated smear or growing of *M. tuberculosis* in any culture medium. The association with each all individual predictors was initially assessed by calculating bivariate Odds ratios. To evaluate the association with the variables included in the score above, we performed multiple logistic regression analysis including all the variables with p values less than 0.2 in the bivariate analysis in addition to the variables present in the score. The later were preserved in the final model independently of the significance attained, while other variables were dropped by backward elimination. The goodness of fit of the model was assessed by means of Hosmer and Lemeshow test.

We calculated for all included patients the total score and classified them according to the corresponding degree of probability of having SNPT (low, intermediate or high). The prevalence of tuberculosis in each of these three groups was established and the likelihood ratios corresponding to the score cut-off points were calculated as well as the corresponding 95% confidence intervals. We further stratified this analysis by previous history of tuberculosis, since lung scarring can cause permanent apical infiltrates as well as chronic cough, even in the absence of active tuberculosis. Additionally, ROC curve analysis [20] was performed. All analyses were done in STATA version 8.2 [21].

## Results

A total of 780 smear negative pulmonary tuberculosis suspects were recruited. 66 were excluded due to obvious other pathology and 30 due to inability to produce sputum. Out of the 684 patients included, 21 had incomplete information (7 had lost or contaminated cultures and 14 incomplete clinical information), and 663 were analyzed. Due to stock shortage between September 2005 to January 2006 and between July and September 2006, MGIT could not be performed for 130 of them. The results can safely be assumed to be missing at random.

184 participants (27.8%) had a final diagnosis of SNPT. 182 of them had at least one positive culture for *M.tuberculosis* and 59 a positive concentrated sputum smear. 2 of these 59 patients had negative cultures.

184 participants (27.8%) had a final diagnosis of SNPT out of whom 182 were diagnosed based on a positive culture for *M.tuberculosis* Positive sputum concentrate was positive in 59 patients with two of them having negative cultures. In addition to age, hemoptysis, weight loss and expectoration -variables included in the score- fever and previous history of tuberculosis were associated with SNPT in bivariate analysis (Table 1). On the other hand, miliary infiltrate was not. In multivariate analysis, fever was not a significant predictor but previous history of tuberculosis (OR = 0.33; 95%CI 0.21–0.51) remained significant. The adjusted Odds Ratios for the variables included in the evaluated score [17] were significantly different from 1, except for miliary infiltrate (Table 2). Goodness of fit for the model was appropriate (p = 0.66; Hosmer and Lemeshow test). Notwithstanding, in the subgroup of patients without previous tuberculosis the only two patients with miliary infiltrate had a final diagnosis of tuberculosis.

**Table 1:** Sociodemographic, clinical and radiological variables in smear negative patients with and without pulmonary tuberculosis. Lima, Peru, 2005–2008.

	Total	PTB	No PTB	OR	P value
	(n = 663)	(n = 184)	(n = 479)		
Male Sex	370(55.8)	109(59.2)	261(54.57)	1.21 (0.86–1.71)	0.27
Age (s.d)	41.4 (17.2)	36.3 (15.8)	43.3 (17.3)	0.97 (0.96–0.98)	<0.01
Fever	43(6.5%)	23(12.5)	20(4.2)	3.64 (1.51–8.80)	<0.01
Hemoptysis	205(30.9)	77(41.9)	128(26.7)	1.97 (1.38–2.82)	<0.01
Productive cough	443(66.8)	109(59.2)	334(69.7)	0.63 (0.44–0.90)	<0.01
Weight loss	417 (62.90)	132(71.7)	285(59.5)	1.73 (1.19–2.50)	<0.01
Previous history of TB	239(36.1)	45(24.5)	194(40.5)	0.48 (0.32–0.70)	<0.01
TB contact	314(47.36)	92(50)	222(46.4)	1.16 (0.82–1.63)	0.40
HIV infection†	98(24.0)	24(19.5)	74(26.0)	0.69 (0.41–1.16)	0.16
Alcoholism	41 (6.18%)	11(6.0)	30(6.3)	0.95 (0.47–1.94)	0.89
Abnormal CXR	538(81.2)	167(90.8)	371(77.5)	2.86 (1.66–4.92)	<0.01
Miliary infiltrate	5(0.8)	3(1.6)	2(0.4)	3.95 (0.66–23.85)	0.13*
Apical infiltrate	379(57.2)	133(72.3)	246(51.4)	2.47 (1.71–3.57)	<0.01

Values in parenthesis are percentages unless otherwise indicated.

†Results available for 408 patients who accepted voluntary counselling and testing.

*Based on Fisher exact test.

PTB  =  Pulmonary tuberculosis.

**Table 2:** Adjusted Odds Ratios for predictive variables included in the evaluated score [17] for smear negative pulmonary tuberculosis. Lima, Peru, 2005–2008.

	No Previous History of Tuberculosis			Previous History of Tuberculosis		
Variable	OR	95% CI	P value	OR	95% CI	P value
**Hemoptysis**	3.23	1.98–5.26	<0.01	0.55	0.26–1.16	0.12
**Age>45**	0.42	0.25–0.72	<0.01	0.44	0.21–0.91	0.03
**Weight loss**	1.77	1.07–2.93	0.03	1.74	0.84–3.59	0.13
**Expectoration**	0.61	0.38–1.00	0.05	0.89	0.39–2.03	0.79
**Apical infiltrate**	4.04	2.50–6.55	<0.01	1.72	0.75–3.95	0.20
**Miliary infiltrate**	NA*			2.04	0.17–23.91	0.57

*Not-entered in the model since the only two cases with miliary infiltrate and no previous history of TB had a final diagnosis of pulmonary tuberculosis.

**Figure 1:**
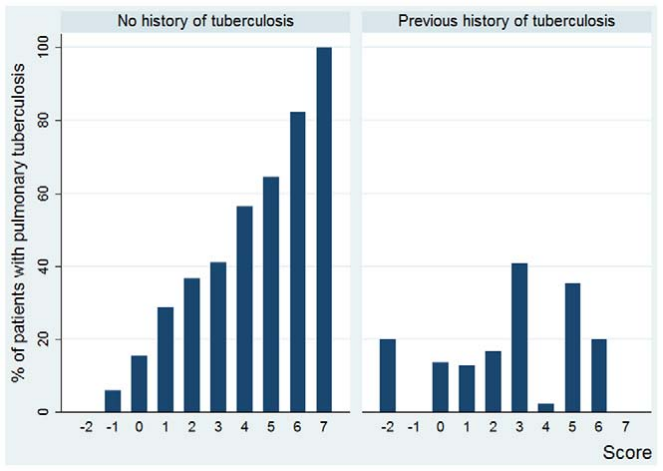
Proportion of patients with smear negative pulmonary tuberculosis by score values in subjects without and with previous history of tuberculosis.

Table 3 shows the prevalence of SNPT in each of the strata based on the score results and our previously suggested cut-off points [17]. The score performed substantially better in patients without history of previous tuberculosis (n = 424): in this group, the prevalence of tuberculosis was 5.1% for low probability patients, 31.7% for intermediate probability patients and 72% for high probability patients. For patients with previous history of tuberculosis, the corresponding percentages were 8.3%, 18% and 29.6% respectively. Figure 1 shows the prevalence of tuberculosis for each value of the score in patients with and without previous history of tuberculosis.

**Table 3:** Prevalence of Smear Negative Pulmonary Tuberculosis (SNPT) according to score and history of previous tuberculosis, Lima, Peru, 2005–2008.

	Score<0 (low probability)		Score 0–4 (intermediate probability)		Score≥5 (High probability)	
	No previous TB	Previous TB	No previous TB	Previous TB	No previous TB	Previous TB
**Number of suspect patients**	59	12	315	200	50	27
**Number with SNPT**	3	1	100	36	36	8
**% with SNPT**	5.1	8.3	31.7	18.0	72.0	29.6

**Figure 2:**
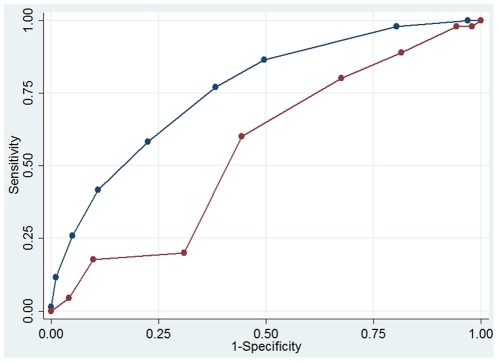
Comparison of ROC curves for the score in patients without (blue) and with (red) previous history tuberculosis.

The area under the ROC curve (AUC ROC) was 0.76 (95% CI 0.72–0.80) for patients without and 0.56 (95% CI 0.50–0.62) for patients with a previous diagnosis of tuberculosis (Figure 2). In patients without previous tuberculosis, scores <0 had a negative likelihood ratio (LR) of 0.11 (95% CI 0.04–0.35) and a negative predictive value of 94.9% (95% CI 85.9–98.9) and scores ≥5 had a positive LR of 5.27(95% CI 2.94–9.45) and a positive predictive value of 72.0% (95% CI 57.5–83.8). Of note, scores of ≥6 had a positive LR of 10.9 (3.24–36.9) in patients without history of TB.

## Discussion

Our study was designed to validate a scoring system developed by Soto et al. [17] for estimating the probability of pulmonary tuberculosis in smear negative suspects. Our main finding is that the score performs well in patients without previous history of PTB, but that it is rather useless in patients who had tuberculosis in the past.

There exist several clinical prediction rules for clinical decision making in tuberculosis and in SNPT in particular [13]–[16], [22], but their utility ought to be assessed in a different sample from the one in which it was derived. To our knowledge, only 1 clinical algorithm has been evaluated independently [22], apart from the score assessed in this study. In addition, most prediction rules derived in resource constrained countries were developed without relying on an adequate reference standard and some even used clinical criteria only[23], [24].

Culture is still considered the reference for diagnosis of tuberculosis [12], but conventional solid cultures lack sensitivity [1], [5], [7].We have used, besides the conventional solid media, two different liquid culture media to improve isolation of *M. tuberculosis* and applied a composite reference standard (positivity in an additional concentrated sputum smear and/or any of 3 types of culture). We did not culture a second sputum sample since its incremental diagnostic yield is poor [25]. One of the liquid culture media was not available during 2 short periods, but this information is “missing at random” and should not bias our results. Although there is theoretically a possibility of false negatives, we are confident that their number must be small and should not invalidate the results of our study.

A limitation of our study is the setting, tertiary level hospitals. This could affect the wider applicability of our findings, but it reflects utilisation patterns and current clinical practice, certainly in large parts of Latin America, where patients with clinical suspicion of pulmonary tuberculosis and negative smears are usually referred to higher level facilities to establish a diagnosis with additional tests and/or make treatment decisions based on expert opinion [26]. However, the presence of diseases such as COPD amongst TB suspects may be higher at referral level. The same holds for HIV co-infection rates. This could influence the performance of the score and should be further evaluated before application in primary care settings.

It is important to stress that the score should not replace clinical judgement but is intended to be an aid to clinical decision making. It is not a diagnostic test but permits to estimate the probability that a suspect patient has smear negative pulmonary tuberculosis. This approach of using 2 cut-off points and 3 categories is considered appropriate when a single cut-off point does not discriminate appropriately between the two disease states of interest [27] and has also been used by other authors [28].The high positive predictive value in patients with a score ≥5 justifies TB treatment initiation, certainly if further confirmatory tests are not readily available. For patients with a score <0, the negative predictive value permits to, at least a “wait and re-evaluate later” approach. We believe this would result in a reduction of diagnostic delays for patients and in a more rational use of constrained diagnostic resources for patients with intermediate to high probabilities of TB. A formal economical evaluation should confirm this, but was outside of the scope of the present study.

We found a higher proportion of subjects in the category with intermediate probability of SNPT than in the original study [17]. Different performances of scores in the derivation and in a validation population are normally expected and a reason why clinical prediction rules must be assessed before being widely applied [29]. Furthermore, a shift in an inclusion criterion (cough >1 week were included in the derivation study against more than two weeks in the present one) may have eliminated patients with low probability of tuberculosis. On the other hand, SNPT suspects with a likely diagnosis of PTB (in particular when a CXR revealed a typical pattern) were probably not referred and hence not included in our study. In practice, the score will be more useful in less selected patient populations, when a substantial proportion of suspects can be assigned to the high or low PTB probability categories. Factors that drive patient selection will always also determine this proportion, and the development of a rapid and inexpensive complementary diagnostic test is, ultimately, the only way forward.

All the variables in the score showed a strong statistical association with SNPT in the bivariate and multivariable analysis, except for miliary pattern in the CXR. This pattern was present in only5 patients included and the two patients with a miliary pattern and negative culture had a final diagnosis of tuberculous sequela. These variables, besides others, have also been identified in previous studies as predictors of SNPT [4], [14], [15], [30], [31]. Our scoring system uses comparable predictive findings to Mello's logistic regression based score [15], the only other clinical prediction rule for SNPT developed in a Latin-American setting, that includes presence of typical X-ray signs, expectoration, weight loss and age. Both scores are quite different from scores derived in African populations [32], [33] –where HIV co-infection are much higher- and in developed countries[16], [34]–[36] –where TB incidence rates are much lower. The former include variables like lymphadenopathy or low haematocrit in the former and the latter immigrant status, BCG vaccination and contact history. These signs and symptoms seem less useful in our setting, which underscores the need for local adaptation and validation of clinical prediction rules.

The evaluated clinical score [17] is a useful tool for assessing, in Peru and possibly in Latin-America, the probability of pulmonary tuberculosis in suspects without previous history of PTB. It can support the decision of treatment initiation or deferral in patients with high or low score based probabilities and indicates the need for further work-up in the group with intermediate probabilities. The adoption of locally validated clinical prediction rules can reduce variability in assessment and management of smear negative tuberculosis. It should become an important subject of operational research in all resource constrained settings.
